# Effectiveness, Safety, and Barriers to Early Mobilization in the Intensive Care Unit

**DOI:** 10.1155/2020/7840743

**Published:** 2020-11-26

**Authors:** Gopala Krishna Alaparthi, Aishwarya Gatty, Stephen Rajan Samuel, Sampath Kumar Amaravadi

**Affiliations:** ^1^Department of Physiotherapy, College of Health Sciences, University of Sharjah, Sharjah, UAE; ^2^Department of Physiotherapy, Kastruba Medical College Mangalore, Manipal Academy of Higher Education, Mangaluru, Karnataka, India; ^3^Department of Physiotherapy, College of Health Sciences, Gulf Medical University, Ajman, UAE

## Abstract

**Purpose:**

Patients admitted to the intensive care unit (ICU) are generally confined to bed leading to limited mobility that may have detrimental effects on different body systems. Early mobilization prevents or reduces these effects and improves outcomes in patients following critical illness. The purpose of this review is to summarize different aspects of early mobilization in intensive care.

**Methods:**

Electronic databases of PubMed, Google Scholar, ScienceDirect, and Scopus were searched using a combination of keywords. Full-text articles meeting the inclusion criteria were selected.

**Results:**

Fifty-six studies on various aspects such as the effectiveness of early mobilization in various intensive care units, newer techniques in early mobilization, outcome measures for physical function in the intensive care unit, safety, and practice and barriers to early mobilization were included. *Conclusion:* Early mobilization is found to have positive effects on various outcomes in patients with or without mechanical ventilation. The newer techniques can be used to facilitate early mobilization. Scoring systems—specific to the ICU—are available and should be used to quantify patients' status at different intervals of time. Early mobilization is not commonly practiced in many countries. Various barriers to early mobilization have been identified, and different strategies can be used to overcome them.

## 1. Introduction

Patients with critical illness are patients with a life-threatening disease or trauma. Such patients are at an increased risk of developing serious complications from their condition [1]. They are admitted to the intensive care unit (ICU) and are usually confined to bed which could negatively affect their mobility [2]. Prolonged immobilization, mechanical ventilation, and sedation in the course of critical illness have been associated with restricted joint mobility, muscle weakness, pressure sores, critical illness neuropathies or ICU-acquired weakness, deep vein thrombosis (DVT), long duration of mechanical ventilation, cognitive impairments, and psychological disturbances [2, 3].

The damaging effects of bed rest are not only limited to the musculoskeletal system but also affects other body systems. Changes noticed within the cardiac system include tachycardia, postural hypotension, decreased peak uptake of oxygen, stroke volume, and cardiac output caused due to fluid loss [4]. A decrease in ventilatory volume and secretion clearance is caused due to a supine position, resulting in atelectasis and pneumonia [2, 4]. Residual problems which include reduced physical function and decreased quality of life are frequently experienced by critical illness survivors [5].

Earlier, bed rest was considered as a treatment for critical illness, but it also had its harmful effects [6, 7]. In 1899, Ries found that bed rest could lead to ill effects in the postoperative period, whereas the length of hospital stay could be shortened from days or weeks to hours using early mobilization [8]. In the following years, similar results were seen in patients who underwent other surgeries and in females in the postnatal period [9]. Also, conferences and peer-reviewed journals addressed the topic of ill effects due to rest in bed [10]. Eventually, the advantages of early mobilization in mechanically ventilated patients were specified in studies [11, 12]. Early mobilization has been tried out as a form of rehabilitation since the early nineteenth century and with a wide array of reported findings of the approach that give it a robust foundation in therapeutic rehabilitation [10].

Early mobilization is the early application and intensification of physical rehabilitation given to patients with critical illness, commenced within the initial two to five days of critical illness [13]. It includes activities such as in-bed mobility activities, range of motion exercises, sitting, standing, transfers, and gait training [4]. However, there is no agreed definition for early mobilization in mechanically ventilated patients, and what activities constitute it are poorly understood [14]. Early mobility in the ICU has been proposed to limit or prevent physical and cognitive dysfunction and provide various benefits [13, 15]. Increase in regional ventilation, perfusion, diffusion, tidal volume, minute ventilation, the efficiency of respiratory mechanics, pulmonary immune factors, mucociliary transport, and airway clearance and decrease in airflow resistance are the changes seen in the pulmonary system [16].

Cardiovascular system changes include an increase in venous return, stroke volume, heart rate, myocardial contractility, cardiac output, coronary perfusion, circulating blood volume, peripheral blood flow, chest tube drainage, and peripheral tissue oxygen extraction. Neurological effects include an increase in level of consciousness and stimulus to breathe. Increased urinary output due to an increase in glomerular filtration is seen [16]. Involving patients in early mobilization may have positive effects such as reduction in muscle atrophy, delirium, and duration of mechanical ventilation, shorter length of ICU stay, enhanced physical function, and quality of life [17].

Considering the benefits of early mobilization, this study aims to review the available evidence on various aspects of early mobilization in the intensive care unit. Understanding its various aspects can aid its implementation in clinical practice and may help in achieving improved patient outcomes.

## 2. Materials and Methods

### 2.1. Study Selection Criteria

Full-text articles on early mobilization of patients—with or without mechanical ventilation—in the intensive care unit, published in the English language from January 2012 to April 2020 were included. Studies conducted in the paediatric intensive care unit, studies on early mobilization outside ICU, study protocols, and poster presentations were excluded.

### 2.2. Literature Search

**Figure 1:**
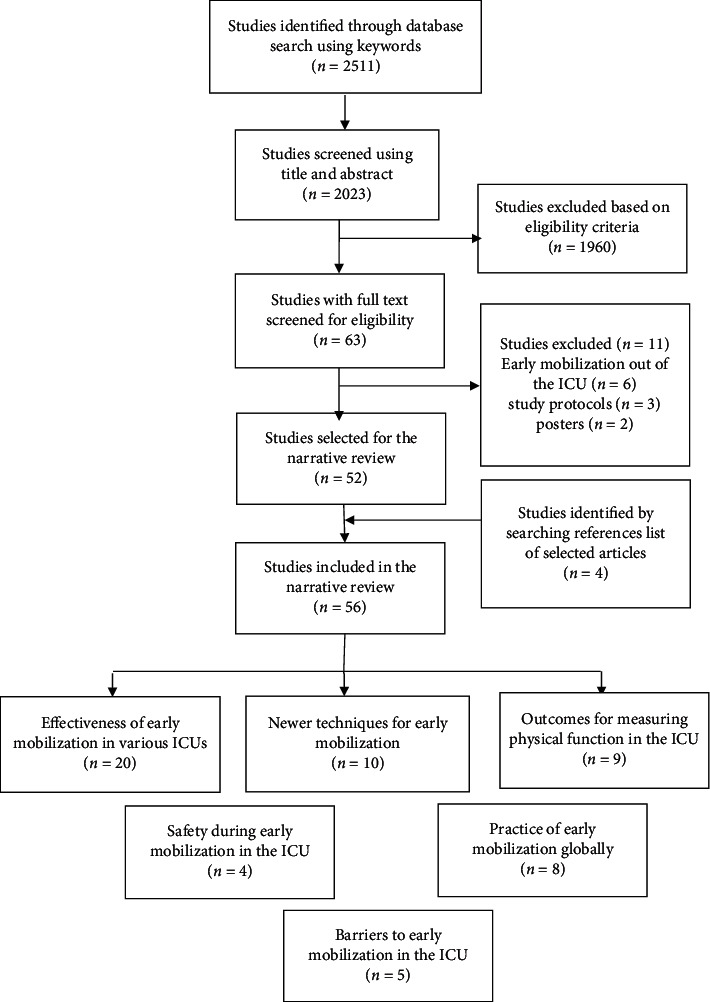
Flow diagram showing data extraction.

Electronic databases of PubMed, Google Scholar, ScienceDirect, and Scopus were searched using combination of keywords “Early Mobilization,” “Early Rehabilitation,” “Intensive Care Unit,” “Critically Ill patients” “Effectiveness,” “Surgical,” “Neurological,” “Cardiac,” “Barriers,” “Outcomes,” “Physical Function,” “Mobility,” “Safety,” “Adverse effects,” “Practice,” “Cycling,” “Electrical Muscle Stimulation,” “Combilizer,” and “Hydrotherapy.” Synonyms were checked to exhaust the possibility of more keywords. The Boolean operator “AND” was used. References lists of the selected articles were manually reviewed. The data extraction is summarized in Figure 1.

## 3. Results and Discussion

Fifty-six studies were included in the review. Out of these, twenty studies were on the effectiveness of early mobilization in various intensive care units, ten studies on newer techniques for early mobilization, nine studies on outcomes for measuring physical function in the intensive care unit, four studies on safety, eight studies about practice, and five studies on barriers to early mobilization.

### 3.1. Effectiveness of Early Mobilization in the Intensive Care Unit

Over the last few years, studies have analyzed the effectiveness of early mobilization on short-term and long-term outcomes in patients with critical illness [17–26].

#### 3.1.1. Short-Term Impact on Outcomes

Zhang et al. [18] found that early mobilization of critically ill patients reduced the incidence of ICU-acquired weakness, improved functional capacity, decreased days on mechanical ventilation, and increased number of patients who could stand and rate of discharge from the ICU.

The systematic review by Doiron et al. [19] reviewed the studies on early mobilization during or after mechanical ventilation versus delayed mobilization or usual care in critically ill patients. It was inconclusive due to the low quality of the included studies.

A meta-analysis carried out by Zang et al. [20] found early mobilization in critically ill to be effective in reducing ICU-acquired weakness and length of ICU stay and in preventing deep vein thrombosis, ventilator-associated pneumonia, and pressure sores. It also improved functional mobility. Similarly, Zhang et al. [17] reviewed the effects of early mobilization for critically ill mechanically ventilated patients and reported that it could improve outcomes such as shortening the duration of mechanical ventilation and decreasing the length of stay in the ICU.

A review by Tipping et al. [21] found that active mobilization in the ICU caused improvement in body function and participation which was measured using muscle strength and walking ability. A systematic review and meta-analysis by Castro-Avila et al. [22] reported that early rehabilitation in patients admitted to the intensive care unit/high dependency unit for more than forty-eight hours improved patients' walking ability at hospital discharge but did not have any effect on muscle strength and functional status.

Most of these reviews had low quality evidence due to reasons such as small sample size, heterogeneity in the population, intervention, and outcome measures. Therefore, good quality studies were suggested to verify the results [17–22]. The need for further studies was advised to determine the effects of different early mobilization protocols and to find out the most effective and safe one [17]. Short-term impacts on outcomes are summarized in Table 1.

#### 3.1.2. Long-Term Impact on Outcomes

Okada et al. [23] conducted a systematic review and meta-analysis to study the effects of delayed versus early mobilization in critically ill adult patients and found no differences between the two groups regarding mortality and health-related quality of life. As the review included studies with limited sample size and heterogeneous definition of interventions, further studies were suggested to confirm these findings. According to Castro-Avila et al. [22], early mobilization did have an impact on the quality of life. Zhang et al. [18] did not find any effect on mortality. Tipping et al. [21] found that early mobilization did not decrease the mortality of patients.

Effects of intensive, twice daily, upright mobilization was studied on mechanically ventilated patients by Amundadottir et al. [24]. They found no difference in outcomes between the twice-daily mobilization and daily mobilization groups at three, six, and twelve months. A randomized controlled trial by Denehy et al. [25] reported no significant difference in outcomes between intervention and usual care groups after a 12-month follow-up. Another randomized controlled trial conducted by Wright et al. [26] concluded that the intervention group, which received a higher dosage of mobilization, did not seem to show improvement in physical outcomes at 6 months compared to standard physical rehabilitation who received usual mobilization. Loss of follow up was one of the important limitations which could have affected the results in both these studies [25, 26].  Table 2 shows the long-term impact of early mobilization on quality of life and mortality.

#### 3.1.3. Effectiveness of Early Mobilization in Subpopulations of Patients with Critical Illness

*(1) Early Mobilization in the Surgical Intensive Care Unit*. In patients undergoing surgeries, especially abdominal and thoracic surgeries' postoperative complications causing significant morbidity and mortality, increased medical consumption, and increased hospital stay are common. Some of these complications include atelectasis, pneumonia, acute respiratory distress syndrome, and deep vein thrombosis [27]. The reasons for these complications are altered respiratory mechanics, reduced lung volumes, respiratory muscle dysfunction, retention of secretions, changes in oxygenation, immobility, and recumbent position postsurgery. Early mobilization can aid in preventing or minimizing these complications [16].

Castelino et al. [27] conducted a systematic review on the effectiveness of early mobilization on postoperative outcomes following thoracic and abdominal surgery. The quality of included studies was found to be poor, and the results were conflicting. The study was inconclusive.

Schaller et al. [28] conducted a randomized controlled trial to find the effectiveness of early goal-directed mobilization in the surgical intensive care unit and found it to be useful as it improved the mobility of the patients at discharge and decreased the length of ICU stay. As the study included only the surgical patients who were mechanically ventilated for more than forty-eight hours, the results of this study could not be generalized to nonsurgical or nonventilated patients.

Zomorodi et al. [29] developed an early mobilization protocol for patients in surgical and trauma ICU. It was found that the protocol was successful and decreased the length of ICU stay. As this was a pilot study, authors suggested that further studies with a larger sample size should be performed to establish the feasibility and efficacy of this protocol.

*(2) Early Mobilization in the Cardiac Intensive Care Unit*. Cardiac surgeries include surgical procedures for pathologies of the heart and have significant effects causing a change in the physiological mechanisms of patients in different ways. This may lead to critical postoperative conditions that require intensive care to establish a functional recovery [30]. Cardiac surgeries present some typical complications such as acute myocardial infarction and low cardiac output syndrome [30, 31]. Some of the other complications are mechanical ventilation for more than forty-eight hours after surgery, acute respiratory distress syndrome, pleural effusion, hypoxemia, acute respiratory failure, phrenic nerve palsy, ventilation-associated pneumonia, cerebrovascular accident, infection at surgical sites, hemorrhage, and changes in the serum electrolytes level [30].

One of the well-established contributing factors to postoperative complications is bed rest or immobility. Despite this, bed rest after surgery was being prescribed for cardiac surgery patients to reduce cardiac overload. Nevertheless, recent evidence shows numerous benefits of early mobilization postsurgery [32]. Studies have shown early mobilization to reduce postoperative outcomes [31, 32].

A systematic review by Santos et al. [32] reported that early mobilization in patients after cardiac surgery prevented postoperative complications, decreased length of hospital stay, and improved functional capacity when compared with no treatment. The most effective protocol could not be found when different techniques and duration of mobilization were compared.

Moradian et al. [31] conducted a randomized controlled trial to study the effect of early mobilization on pulmonary complications after coronary artery bypass graft (CABG) and found a lower incidence of atelectasis, pleural effusion, and improved oxygenation in the intervention group. They suggested that further studies should be performed for identifying appropriate initiating time, frequency, intensity, and duration of early mobilization.

*(3) Early Mobilization in the Neurological Intensive Care Unit*. Acute cerebrovascular accident, subarachnoid, parenchymal and subdural hemorrhage, central nervous system infection, status epilepticus, brain tumors, neuromuscular disorders, and cerebral vascular malformation are common conditions managed in the neurological intensive care unit [33].

They are put on prolonged bed rest for the adequacy of blood flow to the brain resulting in deconditioning and electrolyte imbalance, which augments the already damaging neurological injury [33]. Sympathetic functions are altered, contributing to orthostatic hypotension after long-term bed rest [34]. Patients with severe brain injuries, such as head trauma, large brain infarcts, and subarachnoid hemorrhage may have severe cardiovascular manifestations such as arrhythmias, myocardial ischemia, hypertension, and pulmonary edema. They are also at risk of secondary brain injury because of edema and delayed vasospasm [33, 34].

Early mobilization is considered to be an essential aspect of care, which leads to improved outcomes [35]. Cognitive impairment, hemiparesis or hemiplegia, fluctuating intracerebral pressure and cerebral perfusion, and dislodgement of cerebral monitoring or other indwelling devices can compromise safety during mobilization [33]. Various studies on different neurological conditions have been performed to check the effectiveness of early mobilization [33–38].

Klein et al. [33] conducted a comparative study to assess the effects of early mobilization in improving mobility and clinical outcomes in the neurological ICU and found an increase in the patients' highest level of mobility without causing any severe complications.

Rocca et al. [34] studied the changes in the sympathetic system due to early mobilization with three methods: standard mobilization, gradual postural variations with robot Erigo consisting of a tilting table integrated with leg movement system, and cycling with MOTOmed consisting of an automatic leg mobilization system in supine. They found that both the new methods caused sympathetic stimulation and can be used for early mobilization, but leg movements with MOTOmed caused an increased level of catecholamine, indicating stress, and hence should be used with caution. As the sample size was small and heterogeneous, further studies are required to confirm the results of this study.

Alamri et al. [36] conducted a study to check the effectiveness of an early mobility protocol for patients diagnosed with stroke in the ICU. The patients were divided into three categories, which included unstable and on the ventilator, cooperative and on the ventilator, and cooperative and being weaned off from the ventilator. They were treated with different protocols. Early mobility protocols had positive effects on muscle strength and quality of life. No adverse events occurred; so, they were considered safe to be practiced.

Diserens et al. [37] studied the effectiveness of early mobilization in comparison with delayed mobilization in subjects with moderate to severe acute ischemic stroke and reported an apparent decrease in severe medical complications with early mobilization. Cerebral blood flow on transcranial Doppler and neurological scales showed that the protocol was safe. However, this study was performed on a small sample size with unequal dropouts, and Doppler could be performed only in 60% of the planned instances.

A study was conducted by Bartolo et al. [38] to determine the influence of early mobilization on functional outcomes in patients with severe acquired brain injury and was found to have a positive influence on clinical and functional recovery of the patients.

### 3.2. Effectiveness of Newer Techniques

Various techniques such as electrical muscle stimulation and cycling are being used in early mobilization. Studies have been performed to check the effectiveness of these techniques [39–48].

#### 3.2.1. Electrical Muscle Stimulation

Electrical muscle stimulation (EMS) can be used as a substitute for the reversal of muscle weakness and deconditioning as applying EMS around the muscle fibers and at the neuromuscular junction generates contractions that prevent atrophy of muscles, improve circulation of blood, and alleviate the effects of long periods of immobility without overloading the cardiovascular system. These benefits may persevere for up to four to six weeks after the completion of the treatment [39, 40].

Falavigna et al. [39] conducted a randomized clinical trial to assess the effects of early EMS on the ankle joint range of movement and circumference of the thigh and legs in critically ill mechanically ventilated patients. It showed that EMS was effective in preserving amplitude of the ankle joint movement, increasing mobility and function, but the strength and cross-sectional area of the muscle stimulated did not increase. This could have been due to low intensity and duration of stimulation. Neuromuscular stimulation (NMES) was found to be effective in preserving the thickness of the chest and abdominal muscles in critically ill patients in a study by Acqua et al. [41].

Fischer et al. [42] studied the effect of neuromuscular stimulation in patients after cardiothoracic surgery and concluded that it did not affect the muscle layer thickness and functional outcomes but contributed to higher regain in muscle strength during the ICU stay. Also, a review by Baron et al. [40] suggested that neuromuscular stimulation in the intensive care unit has positive effects and is safe to be used.

#### 3.2.2. Cycling

Effect of cycle ergometry in early mobilization postcardiac surgery was studied in a randomized controlled trial by Gama Lordello et al. [43]. They concluded that it was safe to use but did not cause any significant difference in independent physical activity in the intervention group when compared to a standard care group.

Machado et al. [44] assessed the effects of passive cycling along with conventional physical therapy on muscle strength of peripheral muscle, number of days on a mechanical ventilator, and length of hospital stay in patients admitted to the ICU. Early mobilization using passive cycling improved peripheral muscle strength in mechanically ventilated patients with no significant changes in the number of days on the mechanical ventilator or length of hospital stay.

The effects of early mobilization using a bedside cycle ergometer in addition to conventional physical therapy were evaluated by Santos et al. [45] in a randomized controlled trial. Thickness and architecture of the quadriceps were evaluated in critically ill patients receiving invasive mechanical ventilation. No significant difference was found in these outcomes.

#### 3.2.3. Cycling and Electrical Muscle Stimulation

Fossat et al. [46] found that early in-bed cycling exercise and electrical muscle stimulation for quadriceps did not cause any significant change in global muscle strength at discharge from the ICU when compared to usual care. In addition, there were no significant differences in secondary outcomes such as the number of ventilator-free days, ICU mobility score, or quality of life at 6 months.

#### 3.2.4. Sara Combilizer

The Sara Combilizer is a combination of a tilt table and chair, which can be made completely horizontal to allow transfer through a sliding board and also allows standing positions to be attained. McWilliams et al. [47] assessed the effectiveness of the Sara Combilizer in facilitating safe and early mobilization of critically ill patients and found a reduction in time required for mobilization. It may be a beneficial adjunct to early mobilization protocols.

#### 3.2.5. Hydrotherapy

Felten-Barentsz et al. [48] conducted a study to determine the feasibility and safety of hydrotherapy in critically ill patients who were mechanically ventilated. An individualized tailored program, which could include standing, walking, backstroke swimming, and moving upper limbs, was used. Any adverse events or contamination of pool water were noted. They found hydrotherapy to be feasible and safe and also concluded that further studies need to be performed to assess its cost-effectiveness and benefits.

### 3.3. Outcome Measures for Assessment of the Effectiveness of Early Mobilization

An outcome measure is any characteristic or quality measured to assess a patient's status. They are used to objectively determine the baseline function of a patient at the beginning of treatment and to determine the progress and treatment efficacy [49]. Various studies have used different outcomes for measuring the effectiveness of early mobilization and are mentioned in Table 3 [17–26].

Physical function impairment is a noteworthy problem faced by critical illness survivors. Measuring impairments provide information about the patients' limitations. The selection of the most appropriate measure must be made based on the psychometric properties [50]. In a review, González-Seguel et al. [51] identified sixty physical function measurement instruments under different domains for adult patients admitted to the ICU. Mobility was the most frequent domain to be measured and included 38 instruments. Some of the scoring systems for assessing the effectiveness of early mobilization on mobility in the ICU are mentioned in Table 4 [52–59].

### 3.4. Adverse Effects and Safety during Mobilization

Desaturation, heart rate elevation over 20%, postural hypotension, unplanned extubation, tachypnea, agitation, discomfort, dislodgement of devices, and falls are some of the adverse events in different studies summarized in a systematic review [60].

There is a low frequency of adverse events associated with early mobilization of patients in the ICU (≤4%) and most of them being nonthreatening. Even then, there are possibilities that mobilization may be withheld due to the concern of adverse effects [61]. Assessment before any mobilization session is necessary for the safety of the patient and for minimizing risk due to adverse events. Assessment of whether a patient should be mobilized or not can be aided by objective criteria that ensures safety [60, 61].

A review on safety criteria for starting early mobilization was conducted by Albanaz da Conceição et al. [60]. The safety criteria included parameters that were categorized into groups, which included cardiovascular, respiratory, neurological, and others. It is described in Table 5.

Hodgson et al. [61] developed safety measures for the active mobilization of patients with critical illness who are mechanically ventilated. They used a color-coded system for the criteria. The red signal indicated a significant risk of adverse events. The yellow signal indicated that mobilization was possible but only after measuring risk versus benefit and there could be a potential risk of adverse events. The green signal indicated that active mobilization could be performed with a low risk of adverse events. The red and green signals are mentioned in Tables 6 and 7.

### 3.5. Criteria for Termination of Early Mobilization

Safety criteria also include termination criteria that indicate discontinuation of the early mobilization session and allow the patients to rest [61]. Studies performed by Liu et al. [62] and Perme and Chandrashekar [63] have suggested criteria for termination of mobilization, which are stated in Table 8.

### 3.6. Practice of Early Mobilization Globally

Early mobilization is an intervention that reports positive outcomes, is considered safe to be practiced, and has safety and termination criteria [64–66]. Various studies have developed protocols for early mobilization in different ICUs which have been found to be effective, safe, and practicable. These protocols include different levels of mobilization consisting of activities such as passive or active range of motion exercises, positioning, sitting upright on the bed, sitting on edge of bed, standing, sitting on chair, and walking and are applicable to patients with or without mechanical ventilation [29, 36, 67, 68]. Even then, early mobilization is not practiced commonly. Studies on the practice of early mobilization in different countries are mentioned in Table 9 [65, 69–75].

### 3.7. Barriers to Early Mobilization

The practice of early mobilization is still not common in the clinical setting due to different perceived barriers [76, 77]. Some of these barriers include hemodynamic instability, presence of vascular attachments, altered sleep patterns, safety of the patients, lack of communication and teamwork between various professionals, lack of professionals, inadequate time, delirium, extreme sedation, risk of musculoskeletal injury, and extreme stress at work [13, 78, 79].

Leditschke et al. [80] reported modifiable and nonmodifiable barriers to early mobilization. Modifiable barriers comprised vascular access catheters in a femoral position, sedation management, timing of procedures, agitation, and low Glasgow Coma Score. Nonmodifiable factors comprised hemodynamic instability, respiratory instability, neurologic instability (difficulty in controlling intracranial hypertension), and medical orders. According to Sibilla et al. [70], less-perceived barriers were encountered during passive mobilization when compared to patients who were mobilized actively. Various barriers to early mobilization are mentioned in Table 10 [81, 82].

### 3.8. Strategies to Overcome Barriers

Having an understanding of barriers for early mobilization and developing strategies to overcome those assist professionals in practicing early mobilization as part of daily clinical practice. A study by Dubb et al. [83] merged the available data on early mobilization barriers and strategies to overcome them. A few of them are stated in Table 11.

## 4. Conclusion

Early mobilization is found to have positive effects such as decreasing muscle atrophy, mechanical ventilation duration, length of hospital stay, and increasing functional capacity but do not have an impact on long-term outcomes. Early mobilization—in different intensive care units, namely, surgical, cardiac, and neurological ICU—has been studied and found to be effective. As suggested by most of the systematic reviews, further good quality studies need to be conducted. Also, mobilization protocols need to be compared to find the most effective protocol. Newer techniques such as electrical muscle stimulation, cycling, and hydrotherapy are safe and found to have some positive outcomes. Sara Combilizer can be used safely to facilitate early mobilization. More randomized controlled trials need to be performed to confirm the findings of the existing studies. Outcome measures, specific to the ICU, are available and should be used to quantify patients' status at different intervals of time and to identify achievements due to early mobilization.

Safety criteria, with various physiological considerations, for in-bed and outside bed mobilization along with criteria for termination have been mentioned by various authors and can be used for safe practice. Although early mobilization has benefits, it is not commonly practiced in many countries. Various barriers to early mobilization, which include patient-related, institution-related, and clinician-related barriers have been identified, and different strategies have been used to overcome them to allow the smooth practice of early mobilization.

## Figures and Tables

**Table 1:** Short-term impact of early mobilization on outcomes.

	Studies					
Outcomes	Zhang et al. [18]	Doiron et al. [19]	Zang et al. [20]	Zhang et al. [17]	Tipping et al. [21]	Castro-avila et al. [22]

Muscle strength	No increase	Inconclusive	Prevented weakness	—	Improved	No difference
Complications such as deep vein thrombosis, pneumonia, and pressure sores	—	—	Incidence reduced	—	—	—
Delirium	—	Inconclusive	—	—	—	—
Length of ICU stay	—	Inconclusive	Decreased	Decreased	Could not be analyzed	No difference
Length of hospital stay	—	Inconclusive	Decreased	No difference	—	—
Duration of mechanical ventilation	Decreased	Inconclusive	No effect	Shortened	Could not be analyzed	—
Functional capacity	Improved		Improved	—	—	No effect
Physical function	—	Inconclusive	—	—	—	—
Walking ability	—		—	—	Improved	Improved
Discharge to home rate	Increased		—	—	—	—
Death in ICU	—	Inconclusive	No difference	—	—	—
Mortality at hospital discharge	—	—	—	—	No difference	—

**Table 2:** Long-term impact of early mobilization on outcomes.

Studies	Outcomes	
	Quality of life	Mortality
Okada et al. [23]	No difference between delayed and early mobilization groups	No difference between delayed and early mobilization groups
Zhang et al. [18]	—	No decrease in 28 days mortality rate
Doiron et al. [19]	Inconclusive	—
Tipping et al. [21]	No difference at 6 months	No difference
Castro-Avila et al. [22]	No effect	—
Amundadottir et al. [24]	No difference between intensive, twice-daily mobilization, and daily mobilization groups	No difference between intensive, twice-daily mobilization, and daily mobilization groups
Denehy et al. [25]	No difference at 12 months	—
Wright et al. [26]	No difference at 6 months	—

**Table 3:** Outcome measures for assessing the effectiveness of early mobilization in the intensive care unit.

Outcomes	Outcome measures

Muscle strength	Hand-held dynamometer
	Medical Research Council Score
	Incidence of intensive care unit-acquired weakness (ICUAW) at hospital discharge

Physical function	ICU Mobility Scale
	Surgical intensive care unit optimal mobility score (SOMS)
	Interval scores Physical Function ICU Test (PFIT-s)
	Short Physical Performance Battery score (SPPB)
	Functional Independence Measure (FIM)
	Barthel Index
	Six-minute walk test

Quality of life	36-item Short Form Health Survey (SF-36)
	SF-36 physical health summary score and mental health summary scores

**Table 4:** Outcome measures for assessing the effectiveness of early mobilization on mobility in the intensive care unit.

Author (year)	Outcome measure	No. of items	Total score	Psychometric properties

Tipping et al. [53] (2016)	ICU Mobility Scale	11	0–10	Valid Responsive Acceptable floor and the ceiling effect

Perme et al. [52] (2014) Kawaguchi et al. [54] (2016)	Perme ICU Mobility Score	15	0–32 Higher score—few potential mobility restrictions and decreased assistance Lower score—more potential restrictions to mobility and more assistance needed for mobility	Valid High reliability (*α* > 0.90)

Corner et al. [55] (2014)	Chelsea Critical Care Physical Assessment tool	10	0–50	Valid Limited floor and the ceiling effect

Denehy et al. [56] (2013)	Interval scores Physical Function ICU Test (PFIT-s)	4	0–12	Valid MCID = 1.5points (on interval of 10)

Thrush et al. [57] (2012) Huang et al. [58] (2016)	Functional status score for intensive care unit (FSS-ICU)	5	0–35 Higher the score, better the physical functioning	Valid Responsive Good internal consistency MCID = 2–5

Kasotakis et al. [59] (2012)	Surgical intensive care unit optimal mobility score (SOMS)	5	0–4 Higher the score, better the mobility	Valid Reliable

**Table 5:** Safety measures for early mobilization in the intensive care unit.

Respiratory considerations	Cardiovascular considerations	Neurological considerations	Others

(i) Peripheral oxygen saturation >88% (ii) Respiratory rate >5 bpm (iii) <40 bpm (iv) FiO_2_ < 0.6 (v) PEEP <10 cm H_2_O (vi) Airway protection	(i) Heart rate >40 bpm and <130 bpm (ii) Systolic blood pressure < 180 mm Hg > 90 mm Hg (iii) Mean arterial pressure >60 or <110 mm Hg (iv) No vasoactive medications (v) No increase in the dose of vasopressor in the past two hours (vi) No myocardial ischemia (vii) No arrhythmia (viii) No repetition of antiarrhythmic medications	(i) Level of consciousness, no agitation (ii) Not in coma (iii) Following commands (iv) Delirium (v) Intracranial pressure—not elevated	(i) No unstable fracture or bony instability (ii) Not under continuous hemodialysis (iii) No deep vein thrombosis (iv) Body temperature < 38.5° (v) No active bleeding

FiO_2_, fraction of inspired oxygen; PEEP, positive end-expiratory pressure.

**Table 6:** Red signals for active mobilization of mechanically ventilated patients.

● = red signal	Exercise in bed	Exercise outside bed

Percutaneous oxygen saturation <90%		●
High frequency oscillatory mode of ventilation		●
Prone positioning	●	●
Intravenous hypertensive therapy for emergency hypertension	●	●
Bradycardia requiring pharmacological intervention or awaiting pacemaker insertion	●	●
Mean arterial pressure below the target range		●
Dependent rhythm on a transvenous or epicardial pacemaker		●
Stable tachycardia with a ventricular rate >150 bpm		●
Intraaortic balloon pump		●
Extracorporeal membrane oxygen		●
Cardiac ischemia (ongoing chest pain)		●
Unarousable or deeply sedated patient: RASS < −2		●
Very agitated or combative patient: RASS > +2	●	●
Active management of intracranial hypertension and raised intracranial pressure	●	●
Open lumbar drain (unclamped)		●
Uncontrolled seizures	●	●
Unstable/unstabilized major fractures		●
Large exposed surgical wound		●
Known uncontrolled active hemorrhage	●	●
Femoral sheath		

RASS, Richmond Agitation-Sedation Scale.

**Table 7:** Green signals for active mobilization of mechanically ventilated patients.

✓ = green signal	Exercise in bed	Exercise outside bed

Endotracheal tube	✓	✓
Tracheostomy tube	✓	✓
Fraction of inspired oxygen ≤0.6	✓	✓
Percutaneous oxygen saturation ≥90%	✓	✓
Respiratory rate ≤30 bpm	✓	✓
PEEP ≤10 cm H_2_O	✓	✓
Mean arterial pressure more than the lower limit of target range while receiving no support or low level of support	✓	✓
Stable underlying rhythm with a transvenous or epicardial pacemaker	✓	✓
Femoral intraaortic balloon pump	✓	
Ventricular assist device	✓	✓
Extracorporeal membrane oxygenation: femoral or subclavian	✓	
Pulmonary artery catheterization or other continuous cardiac monitors	✓	
Known or suspected severe aortic stenosis	✓	
Drowsy, calm, or restless patient: RASS −1 to +1	✓	✓
Delirium tool negative	✓	✓
Delirium tool positive and able to obey simple instructions	✓	
Craniectomy	✓	
Lumbar drain (unclamped)	✓	
Acute spinal cord injury	✓	
Subarachnoid bleed with unclipped aneurysm	✓	
Large open surgical wound	✓	
Suspicion or increased risk of active hemorrhage	✓	
Intensive care unit-acquired weakness	✓	✓
Continuous renal replacement therapies	✓	✓
Arterial and venous femoral catheters	✓	✓
Other attachment and drains	✓	✓

PEEP, positive end-expiratory pressure; RASS, Richmond Agitation-Sedation Scale.

**Table 8:** Criteria for termination of mobilization.

(i) Tachycardia (>140 beats/min)
(ii) Bradycardia (<50 beats/min)
(iii) Arrhythmias
(iv) Hypertension—systolic blood pressure >180 mm Hg
(v) Hypotension—systolic blood pressure < 80 mm Hg
(vi) Symptomatic orthostatic hypotension
(vii) Mean arterial pressure <60 or >110 mm Hg
(viii) Oxygen saturation < 88%
(ix) Asynchrony with mechanical ventilation
(x) Abnormality in respiratory rate—>40 breaths/min or <5 breaths/min
(xi) Significant use of accessory muscles
(xii) Significant chest pain
(xiii) Excessive pallor or flushing of the skin
(xiv) Extreme fatigue
(xv) Patient's intolerance or request to stop
(xvi) Hemorrhage and unexpected removal of medical devices such as the chest tube, endotracheal tube, feeding tube, abdominal drain, urinary catheter, arterial catheter, hemodialysis catheter, or venous catheter

**Table 9:** Practice of early mobilization.

Study (year)	Study design	Place	Population	Conclusion

Timenetsky et al. [69] (2020)	1-day point prevalence study	Brazil	348 adult patients with more than 24 h of ICU stay (24 mixed ICU, 1 surgical ICU, and 1 medical ICU)	High prevalence of mobilization activities in critically ill patients Not much active mobilization in mechanically ventilated patients

Sibilla et al. [70] (2017)	Point prevalence study	Switzerland	161 mechanically ventilated patients from 35 ICUs	Only 33% of the mechanically ventilated patients actively mobilized

Nydahl et al. [71] (2014)	1-day point prevalence study	Germany	Mechanically ventilated patients	Three quarters of the patients not mobilized out of bed

Berney et al. [72] (2013)	One-day point prevalence study	Australia and New Zealand	514 patients admitted to the intensive care unit from 38 ICUs	Low patient mobilization on that day

TEAM study investigators [65] (2015)	Cohort study	Australia and New Zealand	192 mechanically ventilated ICU patients from 12 ICUs	84% of the physiotherapy sessions did not include early mobilization

Leong et al. [73] (2017)	Cross-sectional survey on early mobilization of mechanically ventilated patients	Malaysia	186 nurses working in adult critical care units of University Malaya Medical Centre (UMMC), a 1200-bed referral centre	Mobilizing patient three times and above per shift was reported by 75% of nurses. 47.7% reported that they only performed passive range of motion to mechanically ventilated patients. 29.5% reported that they only provide active ROM for their patient. 72% nurses reported that they had not gone through patient mobilization training

Bhat et al. [74] (2016)	Cross-sectional survey	India	82 physiotherapists working in neurological intensive care units of India	97.6% participants reported that patients received mobilization in some form. Mobilization in various forms practiced in the neurological ICUs of India. Less availability of physiotherapists on weekends and night hours.

Chawla et al. [75] (2014)	Survey	India	659 physicians of the Indian Society of Critical Care Medicine and the Indian Society of Anesthesiologists who worked full time or part time in intensive care	High awareness of benefits of early mobilization and low implementation

**Table 10:** Barriers to early mobilization.

Author (year)	Reported barriers
Anekwe et al. [81] (2017)	*Perceived patient level barriers*
	(i) Medical instability
	(ii) Risk of dislodgement
	(iii) Excessive sedation
	(iv) Endotracheal intubation
	(v) Cognitive impairment
	(vi) Inadequate analgesia
	*Perceived institutional level barriers*
	(i) Orders required
	(ii) Lack of equipment
	*Perceived provider level barriers*
	(i) Limited staff
	(ii) Communication among providers
	(iii) Inadequate training
	(iv) Not a priority
	(v) Safety concerns

Costa et al. [82] (2017)	*Patient related*
	(i) Lack of patient's cooperation
	(ii) Patient's instability and safety concerns
	(iii) Patient status issues (fatigue, diarrhea, leaking wound, weight size, confusion, agitation, and death)
	*Clinician related*
	(i) Lack of awareness and knowledge about the protocol
	(ii) Lack of conceptual agreement with guidelines
	(iii) Lack of self-efficacy and confidence in protocol implementation
	(iv) Staff and patient safety concerns
	(v) The perception that rest equals healing
	(vi) Reluctance to follow protocol (due to previous adverse outcomes)
	(vii) Lack of confidence
	(viii) Perceived workload
	(ix) Safety of tubes, wires, and catheters
	*Protocol related*
	(i) Unavailability of protocol
	(ii) Unclear protocol criteria
	(iii) Protocol development cost (money and time)
	(iv) Learning curve (possibility for the clinician to test guideline and observe other clinicians using the guideline easily)
	(v) Lack of clarity as to who is responsible, steps needed to take, and expected standards for protocol implementation
	(vi) Lack of confidence in evidence supporting protocol and guideline developer
	(vii) Lack of confidence in the reliability of screening tools
	*ICU contextual barriers culture*
	(i) Interprofessional team care coordination, communication, and collaboration barriers
	(ii) Lack of leadership/management
	(iii) Interprofessional clinician staffing, workload, and time
	(iv) Physical environment, equipment, and resources
	(v) Staff turnover
	(vi) Low prioritization and perceived importance
	(vii) Scheduling conflicts (i- + -e, patient off, at dialysis, and procedure)

**Table 11:** Strategies for overcoming barriers.

Barriers	Strategies

*Patient-related barriers*	
(i) Hemodynamic instability	(i) Stepwise approach
(ii) Pain	(ii) Pain management before mobilization
(iii) Deep sedation	(iii) Regular assessment, lighter sedation
(iv) Agitation and delirium	(iv) Assessment, antipsychotic medications
(v) Patient denial, lacking motivation	(v) Patient education and encouragement
(vi) ICU equipment and devices	(vi) Portable devices, secure lines, drains, and interdisciplinary teamwork

*Structural barriers*	
(i) Limited staff	(i) Additional staff, independent mobility team
(ii) Lack of protocols and limited guidelines	(ii) Develop protocols, safety criteria
(iii) Limited equipment	(iii) Training for appropriate use of equipment, financial, and the cost analysis model of economic benefit

*Cultural barriers*	
(i) Lack of mobilization culture	(i) Promotion of mobility programs
(ii) Early mobilization, not a priority	(ii) Interprofessional education

*Process-related barriers*	
(i) A dearth of coordination and planning	(i) Regular screening of patients, interprofessional coordination, and planning
(ii) Risks for mobility providers	(ii) Training, appropriate equipment, and mobility team
